# Trends in the dietary patterns of Mexican adults by sociodemographic characteristics

**DOI:** 10.1186/s12937-020-00568-2

**Published:** 2020-05-27

**Authors:** Sandra Pérez-Tepayo, Sonia Rodríguez-Ramírez, Mishel Unar-Munguía, Teresa Shamah-Levy

**Affiliations:** 1 Nutrition and Health Research Center, National Institute of Public Health, Av. Universidad No. 655, Col. Santa María Ahuacatitlán, 62100 Cuernavaca, Morelos Mexico; 2 Evaluation and Surveys Research Center, National Institute of Public Health, Av. Universidad 655, Col Sta Ma Ahuacatitlán, 62100 Cuernavaca, Morelos, Mexico

**Keywords:** Trends, Dietary patterns, Diet quality, Mexican adults, Sociodemographic characteristics, National Health and nutrition survey

## Abstract

**Background:**

Sociodemographic characteristics are associated with the dietary patterns of populations. However, the direction of the association is not consistent among countries: it is contingent on the nutritional transition phase, level of economic development, cultural contexts and both the social and health policies prevailing in each country. The objective of this study was to identify the trends in dietary patterns observed in 2006, 2012 and 2016 among Mexican adults by sociodemographic characteristic.

**Methods:**

To determine and compare dietary patterns, we performed a secondary analysis of dietary and sociodemographic data for adults 20–59 years old. Data were drawn from the 2006 and 2012 National Health and Nutrition Surveys (ENSANUTs) together with the 2016 Half-Way National Health and Nutrition Survey (ENSANUTMC). To estimate the dietary patterns, we used an adapted version of the Healthy Eating Index-2015 (HEI-2015) and a quantile-based regression model to compare the HEI medians by sociodemographic characteristic.

**Results:**

From 2006 to 2016, the quality of the diet of Mexican adults scored under 50 points on a scale of 0 to 100, markedly below the maximum scores for the majority of HEI-2015 components. Diet quality varied according to age, sex, socioeconomic status (SES), area (urban/rural) and region of residence, with the highest quality observed among older individuals (within the 40–59 age group), women, people of lower SES and residents of rural areas, particularly in southern Mexico. Although this trend remained constant overall throughout 2006, 2012 and 2016, specific HEI-2015 components showed an opposite trend by sociodemographic strata.

**Conclusion:**

The diet quality of Mexican adults was suboptimal from 2006 to 2016, with notorious disparities persisting over time among sociodemographic strata. Our results can serve as a basis for formulating recommendations on ways to improve the population diet, where those components diverging the most from adequate scores could be highlighted in public-health messages.

## Background

Analyzing dietary patterns –rather than foods or nutrients- has been recommended as an effective methodology for studying diet and its relationship to health [1]. A priori and a posteriori approaches are commonly used for determining the dietary patterns of populations [2]. The a priori approach relies on diet-quality indices that classify dietary patterns based on their adherence to recommendations regarding the food items and nutrients that are important for health. Meanwhile, the a posteriori approach defines patterns according to food-consumption data, and employs mostly factorial and cluster analyses [1–5]. Analyzing the dietary patterns of populations is important because it allows for identifying the characteristics of diets that contribute to the prevention and treatment of disease. Furthermore, the results of these analyses can serve as a basis for formulating nutritional interventions and public-health policies aimed at improving the diets of populations [6].

Analyzing the dietary patterns of distinct population subgroups is also important: it has been documented that sociodemographic characteristics such as age, sex, socioeconomic status (SES) and place of residence are associated with food consumption and therefore influence dietary patterns [7–9]. However, the direction of the association is not consistent among countries [9]. Differences relate to the stages of nutritional transition [6, 7] and socioeconomic development [10], as well as to cultural factors and to the social and health policies [11] prevailing in each country. For instance, it has been demonstrated that individuals of high SES eat healthier diets in low-income countries, but exhibit less healthy eating patterns marked by greater intake of energy and saturated fat in middle-income countries [9].

In Mexico, dietary patterns are associated with SES and area of residence. For example, in urban areas, the higher the SES, the greater the diversity in dietary patterns including dairy products, cereals, meats, saturated fat, fruits and vegetables. Conversely, a low SES in rural areas is reflected in a dietary pattern based primarily on corn derivatives combined with beans and legumes [12].

Overall, the most recent Half-Way National Health and Nutrition Survey (*ENSANUTMC*, by its Spanish acronym) (2016) revealed that only a relatively small percentage of Mexican adults consumed recommended foods (50 and 42.3% of the population ate fruits and vegetables, respectively), while a large percentage consumed foods not recommended for usual intake (85.3% of the population consumed sweetened non-dairy drinks) [13]. Differences also arose by geographic region. For instance, in northern Mexico, the higher the SES, the greater the consumption of fruits, vegetables and non-processed meats [14].

To the best of our knowledge, very few studies have analyzed the trends of dietary patterns in nationally representative samples of Mexican adults according to sociodemographic strata. Given the dearth of information on the subject, we undertook the present study with a twofold objective: to identify the trends in dietary patterns in 2006, 2012 and 2016, and to analyze the results by sociodemographic strata.

## Methods

### Study population and sample size

Our study consisted of a secondary analysis of dietary and sociodemographic information pertaining to Mexican adults between the ages of 20 and 59 years. We used data from the 2006 and 2012 National Health and Nutrition Surveys (*ENSANUTs*, by their Spanish acronym) and from the 2016 *ENSANUTMC*. Based on the same methodology and design, these surveys furnish comparable data. Among other common characteristics, they are nationally representative, they stratify data by area of residence (urban/rural) and they employ probabilistic, multi-stage and cluster sampling methods. A more detailed description of their methodology has been published elsewhere [15–17].

The 2006 *ENSANUT* was conducted from October 2005 to April 2006, the 2012 *ENSANUT* from October 2011 to May 2012, and the 2016 *ENSANUTMC* from May to September 2016. Our study sample included 14,040 respondents from the 2006 *ENSANUT*, 2027 from the 2012 *ENSANUT* and 5729 from the 2016 *ENSANUTMC*.

### Study variables

#### Sociodemographic variables

Our analysis considered five sociodemographic variables: sex, age, area of residence, region of residence and SES. 1) Sex was registered as female/male. 2) Age was calculated in years, both continuous and categorical. We classified participants into two age groups, 20–39 and 40–59 years, as previous studies using diet-quality indices found that young adults ate poorer-quality diets than older adults, and diet quality bore a positive relation to age in all cases analyzed [18, 19]. 3) In line with the three surveys in question, area of residence was defined according to the number of inhabitants: < 2500 for rural and ≥ 2500 for urban localities [15–17]. 4) Region of residence was based on the official Mexican classification, whereby the 32 federal entities are divided into four groups according to their geographic location: North, Center, South and Mexico City along with its metropolitan area [20]. 5) Finally, SES has been consistently used by the ENSANUTs as an index of household wellbeing. It is constructed by analyzing key components related to household characteristics and ownership of household items. The results provide a standardized variable categorized into SES tertiles (low, medium and high). SES distribution in our study was relative to each survey year [21, 22].

#### Dietary information

The three surveys analyzed collected dietary information through a semi-quantitative food-frequency questionnaire (SFFQ) with a seven-day recall previously validated for estimating energy and nutrient intake in adults [23]. Dietary and sociodemographic data were gathered by means of interviews, in conformity with the Manual of the Center for Nutrition and Health Research at the National Institute of Public Health (*INSP* by its Spanish initials) for administering questionnaires and standardizing interviewers [24].

To analyze the dietary data, we adopted the cleaning criteria used by the surveys for each year analyzed and followed the methodology developed by Rodríguez-Ramírez (2009) and Ramírez-Silva (2016) [25, 26]. In general terms, for the 2016 data, we used the commonalities of the 2012 cleaning methodology. Basically, we followed two stages: first, we identified implausible data in terms of grams of ingested food, and excluded individuals who consumed one or more food items three standard deviations beyond the mean amount of ingested grams. During the second stage, we defined plausible data for energy and nutrients taking into account adult nutritional requirements.

For the three survey years, we based the exclusion criteria on minimum-energy-consumption data [26]. In accordance with the Mifflin-St Jeor formulas for overweight and obese individuals ≥19 years old, we first calculated the basal metabolic rates (BMRs) of participants and then estimated the ratios of their total energy intake (TEI) with respect to their BMRs (TEI/BMR). Individuals with a < 0.5 quotient were excluded from our analysis. To identify individuals with valid weight and height data, we adapted the criterion of the World Health Organization (WHO) for determining plausible body mass index (BMI) data (< 10 or > 58 kg/m^2^). Finally, we excluded individuals with incomplete sociodemographic data as well as pregnant and lactating women. We omitted the latter because their energy requirement was not comparable with that of other women.

For the 2006 ENSANUT, the sample with dietary data included 16,494 adults. Among these, we excluded 596 (3.6%) with implausible energy and macronutrient data, 26 (0.1%) with implausible BMI data, 55 (0.3%) with incomplete sociodemographic data, 958 (5.8%) with incomplete anthropometric data and 615 (3.7%) with TEI/BMR ratios < 0.5; another 204 (1.2%) were omitted because they pertained to the group of pregnant and/or lactating women.

With regard to the 2012 ENSANUT, the sample with dietary data included 2297 adults. Among these, we excluded 13 with implausible food consumption, 158 with implausible energy and nutrient intake, and 99 for pertaining to the group of pregnant or lactating women. A final 2027 adults with valid dietary data were retained for analysis. As for the 2016 ENSANUTMC, 6188 adults provided dietary data. Of these, we excluded 324 (5.2%) with implausible energy and macronutrient data, and 135 (2.1%) with implausible BMI data.

#### Dietary patterns

We defined dietary patterns using a modified version of the HEI-2015 methodology, an a priori approach validated for measuring diet quality in adults. The HEI-2015, developed by the National Cancer Institute in collaboration with the US Department of Agriculture [27], includes 13 components. Nine are food groups and nutrients that people are encouraged to continue consuming or incorporate into their diets (adequacy components), namely total fruits, whole fruits, total vegetables, greens and beans, seafood and plant proteins, total protein foods, whole grains, dairy products and fatty acids. The four remaining components are food items that people are advised to include only moderately in their diets (moderation components), namely refined grains, sodium, added sugars and saturated fats. Each component is scored on a scale of 0 to 5 or 0 to 10, depending on the portions consumed and according to adult intake recommendations in the US [28]. For analysis, we modified the cutoff points of three components: sodium, added sugars and saturated fats, as the original ones were considered highly permissible for the Mexican population [29, 30]. We aligned our cutoff points with international recommendations [31, 32]; they are listed by component in Additional file 1: Table S1. Finally, we calculated the sum of the 13 components for each participant, obtaining a total score ranging from 0 to 100, where 100 denoted an ideal diet. Calculations were based on the following steps: (a) We classified the foods in each SFFQ survey into the 13 HEI-2015 components according to the Methodology for Users of Food Patterns Equivalents Database (FPED 13–14) [33]. In the case of foods requiring preparation prior to consumption, we broke down the ingredients by food group (component). Several foods in the 2012 *ENSANUT* had been grouped or omitted in the 2006 *ENSANUT* SFFQ; therefore, we standardized these differences based on the foods in the 2012 and 2016 surveys. More specifically, we allocated nutritional values proportional to the number of foods broken down or included in 2012. (b) We calculated the following nutrients: sodium, fatty acids, added sugars and saturated fats, based on the nutritional contents of the food items. The contents were estimated according to the food composition database compiled by *INSP* researchers. (c) We identified the equivalent portion of each food based on the FPED portions guide. A number of foods consumed in Mexico such as *nopal* and *nixtamalized tortilla* were not included in the guide; we therefore allocated them to the corresponding food group using the predetermined equivalent portions indicated in the 2012 *ENSANUT* SFFQ. As corn *tortillas* vary depending on the manner in which the corn is treated, we classified them as a whole cereal based on the relationship between their fiber and total carbohydrate contents [34]. (d) We estimated the score of each component by participant, assigning it a number from 0 to 5 or 0 to 10, according to the intake portions in the HEI-2015 [35] and those established for three of the components in the Mexican population. We determined intermediate values using a rule of proportion or the rule of three. (e) Lastly, we obtained a final score by adding up the scores of the 13 components for each participant.

### Statistical analysis

We developed descriptive statistics including the mean and the 95% confidence interval of each HEI-2015 component for each survey year and each sociodemographic characteristic. We also used a quantile regression model, adjusting for the survey years and sociodemographic variables, in order to compare the HEI-2015 scores (totals, medians and by component) in the three survey years of interest and by sociodemographic strata. A *p*-value < 0.05 was established to detect significant differences among the categories of variables analyzed. All analyses were carried out using the SVY module in STATA version 13.0 statistical software [36].

## Results

We assessed the dietary and sociodemographic data of adults aged 20–59 from the three above-mentioned *ENSANUTs* in Mexico. All three surveys included a higher proportion of women than men and a larger number of adults in the 20–39 compared to the 40–59 age group. Approximately 25% of households were of low SES and 70% of the population lived in urban areas, with the majority residing in Central and Southern Mexico (Table 1).

**Table 1:** Sociodemographic characteristics of adults by survey year of the National Health and Nutrition Survey

Characteristics	Survey year		
	2006 *n* = 14,040%(95% IC)	2012 *n* = 2027% (95% IC)	2016 *n* = 5729% (95% IC)
Age			
20–39 y	56.3 (54.7–57.8)	55.5 (51.9–58.9)	58.5 (56.1–60.7)
40–59 y	43.7 (42.1–45.2)	44.5 (41.0–48.0)	41.5 (39.2–43.8)
Sex			
Males	38.7 (37.0–40.3)	47.0 (43.7–50.2)	46.9 (44.5–49.1)
Females	61.3 (59.6–62.9)	53.0 (49.7–56.2)	53.1 (50.8–55.4)
Socioeconomic status			
Low	30.0 (28.2–31.9)	25.6 (23.3–28.0)	22.3 (19.8–24.9)
Medium	35.0 (33.2–36.7)	32.1 (28.6–35.8)	30.5 (28.2–32.9)
High	35.0 (32.8–37.7)	42.3 (38.6–45.9)	47.2 (44.0–50.3)
Area			
Urban	81.1 (79.3–82.7)	76.8 (74.7–78.6)	74.7 (71.6–77.4)
Rural	18.9 (17.2–20.6)	23.2 (21.3–25.2)	25.3 (22.5–28.3)
Region			
North	19.5 (17.2–22.0)	20.9 (19.3–22.6)	26.6 (23.7–29.7)
Center	29.6 (26.4–32.9)	31.3 (29.1–33.5)	30.0 (26.6–33.6)
Mexico City	21.4 (17.2–26.1)	16.8 (14.7–19.1)	15.6 (13.2–18.1)
South	29.5 (26.4–32.6)	31.0 (28.8–33.1)	27.8 (24.7–31.1)

N (expanded n): 2006 = 41,656,737, 2012 = 47,761,244, 2016 = 46,005,557

**Fig. 1:**
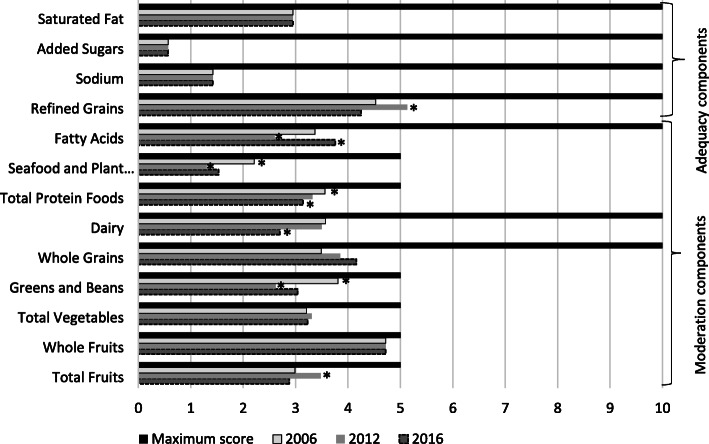
HEI-2015 score (median) by components respect to its maximum score and by survey year. * Different from the other years (*p* < 0.05). Medians adjusted by sociodemographic variables (age, sex, socioeconomic status, area, region)

In comparing each HEI-2015 component score with the highest possible score (Fig. 1), we observed that, in the three survey years analyzed, all components came out lower than the maximum score recommended under the HEI-2015. We also found that the consumption components varied with the survey year; for instance, seafood and plant proteins, total protein foods and greens and beans were higher in 2006 than in 2012 and 2016.

In 2012, Mexican adults consumed more refined grains and total fruits (including natural juices and fruit nectars) but less fatty acids, seafood and plant proteins, greens and beans than in 2006 and 2016. In 2016, more fatty acids but fewer total proteins and dairy products were consumed than in 2012 and 2006; in addition, more whole grains were consumed compared to 2006. No significant differences emerged among the three surveys regarding consumption of the following components: saturated fats, added sugar, sodium, total vegetables and whole fruits. In conducting an analysis without modifying the cutoff points of the three components, we observed some significant differences by survey year for saturated fats and added sugar, as well as high scores –some close to the maximum– for these two components and for sodium (Additional file 2: Fig. S1).

By HEI-2015 component, scores indicated significant differences between adults aged 20–39 and 40–59 years and between men and women (Table 2). In 2006, the adults in the older age group recorded better scores for five components (total fruits, total vegetables, greens and beans, dairy products and seafood and plant proteins) compared to the younger adults, while women achieved better scores for the majority of components (total fruits, total vegetables, greens and beans, whole grains, dairy products, refined grains and sodium) compared to men.

**Table 2:** HEI-2015 component scores (median) by age, sex and survey year^a^

	2006 n = 14,040				2012 n = 2027				2016 n = 5729			
	Age		Sex		Age		Sex		Age		Sex	
Components	20–39	40–59	Male	Female	20–39	40–59	Male	Female	20–39	40–59	Male	Female
Total Fruit	2.8	3.3*	2.3	3.4*	3.4	3.4	3.2	3.6	2.8	2.9	2.4	3.2*
Whole Fruit	4.7	4.7	4.4	4.8	4.7	4.7	4.7	4.7	4.3	4.3	4.1	4.5
Total Vegetables	3.1	3.4*	2.8	3.5*	3.1	3.5*	3.0	3.5*	2.9	3.6*	2.6	3.7*
Greens & Beans	3.8	4.0*	3.8	4.0*	2.5	2.9*	2.6	2.7	2.6	3.4*	2.8	3.1*
Whole Grains	3.3	3.3	2.9	3.6*	4.0	3.8	3.7	4.1	4.2	4.6*	4.2	4.5
Dairy	3.3	3.8*	2.8	4.0*	3.4	3.3	2.8	3.9*	2.8	2.5	2.2	3.1*
Total Protein Foods	3.5	3.5	3.5	3.5	3.3	3.2	3.4	3.2	3.2*	3.0	3.0	3.3*
Seafood and Plant Protein	2.0	2.3*	2.1	2.2	1.3	1.4	1.3	1.3	1.3	1.8*	1.5	1.5
Fatty Acids	3.4	3.3	3.9*	3.0	2.8*	2.2	3.0*	2.2	3.5	3.9*	4.1*	3.3
Refined Grains	4.1	4.3	3.7	4.5*	5.4	4.8	4.9	5.3	4.5	3.8	4.3	4.1
Sodium	1.4	1.9	1.0	2.0*	1.3	1.9*	0.7	2.3*	1.2	1.2	0.4	1.9
Added Sugar	0.2	0.2	0.2	0.2	0.6	0.6	0.6	0.6	0.9	1.1	0.9	0.9
Sat Fat	3.4	3.4	3.4	3.4	2.3	2.3	2.3	2.3	3.6	3.6	4.6*	2.9

^a^ Medians adjusted by sociodemographic variables (age, sex, socioeconomic status, area, region). * Significant difference = *p* < 0.05

In 2012, substantial differences were observed by age for the following components: total vegetables, greens and beans and sodium, with older adults scoring higher than younger adults. In contrast, younger adults registered higher scores for fatty acids. Women had higher scores for total vegetables, dairy products and sodium compared to men. In 2016, the older adults registered markedly higher scores than the younger adults for the following components: total vegetables, greens and beans, whole grains, seafood and plant proteins and fatty acids, while younger adults achieved higher scores for total protein foods. By sex, women registered higher scores for total fruits, total vegetables, greens and beans, dairy products and total protein foods, while men scored higher for saturated fats. In all three surveys, men had higher scores than women for fatty acids, while no differences for whole fruits and added sugar were observed by sex, age or survey year.

**Fig. 2:**
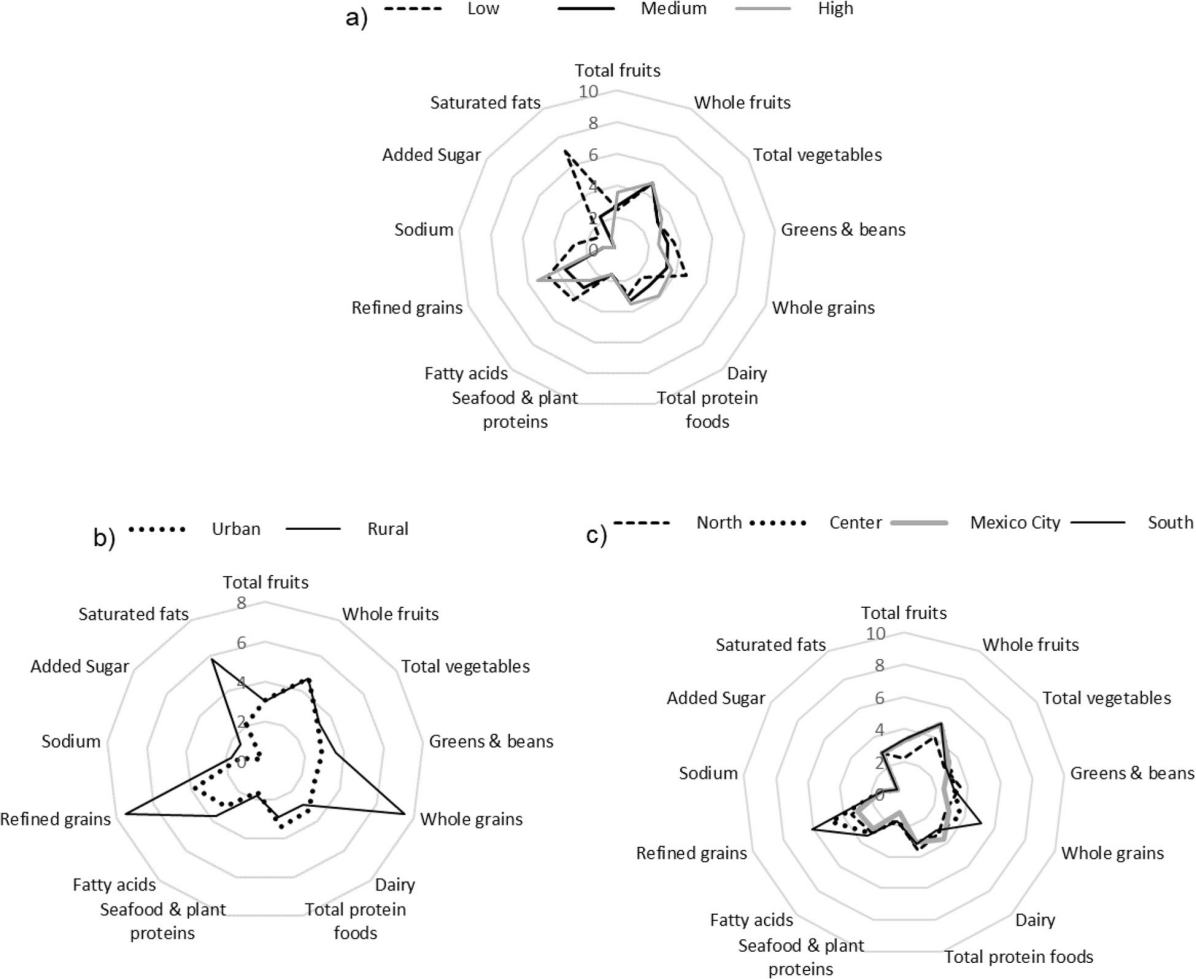
Score of HEI-2015 components by socioeconomic status (a), area (b) and region(c). Medians adjusted by sociodemographic variables (age, sex, socioeconomic status, area and region) and survey year

The scores for the HEI-2015 components by SES, area and region are shown in Fig. 2. As can be observed, groups of lower SES registered significantly higher scores for the following components: legumes, whole grains, fatty acids, sodium and saturated fats, but notably lower scores for dairy products and total protein foods, compared to those of medium and higher SES.

Groups with higher SES achieved significantly higher scores for total fruits, dairy, proteins and refined grains but lower scores for greens and beans, fatty acids and saturated fats, compared to those of medium and lower SES (Fig. 2a).

By area of residence (Fig. 2b), rural residents exhibited substantially higher scores for the majority of components (legumes, whole grains, refined grains, seafood, fatty acids, sodium, and saturated fats) compared to urban residents; however, the latter obtained higher scores for dairy products and total proteins.

By region (Fig. 2c), significantly higher scores were registered in Southern Mexico for whole and refined grains, while the Northern region scored lower than other regions for the following components: total fruits, whole fruits and grains, as well as legumes and total proteins. Finally, Mexico City scored significantly higher than other regions for dairy products but lower in seafood and refined grains.

HEI-2015 scores by sociodemographic characteristic and survey year are shown in Table 3. Adults 20–39 years old achieved significantly higher scores than those 40–59, except in 2012 (*p* = 0.783). Men scored markedly lower than women for all survey years.

**Table 3:** Healthy Eating Index score by sociodemographic stratum and survey year ^1^

Strata	2006 n = 14,040 Median (95% IC)	P	2012 n = 2027 Median (95% IC)	P	2016 n = 5729 Median (95% IC)	P
Age						
20–39	44.0 (43.3–44.7)		43.6 (42.3–45.0)		42.9 (41.9–44.0)	
40–59	47.6 (46.8–48.5)	< 0.001	43.9 (42.5–45.3)	0.783	47.1 (46.3–48.0)	< 0.001
Sex						
Male	44.2 (43.3–45.1)		42.5 (41.1–43.9)		43.1 (42.0–44.2)	
Female	46.4 (45.7–47.1)	< 0.001	44.8 (43.5–46.2)	0.018	46.1 (45.2–47.0)	< 0.001
Socioeconomic status						
Low	47.9 (47.0–48.7)		47.6 (46.2–48.9)		47.6 (45.9–49.2)	
Medium	44.5 (43.6–45.5)	< 0.0001^a^	42.1 (40.2–44.0)	< 0.001^a^	43.2 (41.8–44.6)	0.001^a^
High	44.6 (43.6–45.6)		42.7 (41.0–44.5)		44.2 (43.2–45.2)	
Area						
Urban	43.9 (43.3–44.5)		42.3 (41.2–43.4)		42.9 (42.1–43.8)	
Rural	52.7 (51.6–53.8)	< 0.001	48.5 (46.7–50.4)	< 0.001	49.7 (48.4–51.1)	< 0.001
Region						
North	43.8 (54.0–55.5)		39.5 (37.9–41.1)		41.4 (40.1–42.7)	
Center	47.9 (58.2–59.8)		43.8 (42.1–45.4)		46.2 (44.9–47.6)	
Mexico City	41.3 (52.8–54.4)	< 0.001^abc^	42.5 (39.4–45.5)	< 0.05^acd^	42.4 (40.6–44.1)	< 0.05^abc^
South	47.5 (57.9–59.2)		47.3 (45.6–49.0)		47.4 (40.6–48.8)	
Total HEI-2015 score^2^	45.7 (45.2–46.2)	< 0.001^a^	43.7 (42.8–44.6)	0.029^b^	44.9 (44.2–45.6)	0.079^c^

^*1*^Medians adjusted by age, sex, socioeconomic status, area and region. ^**2**^Medians adjusted by age, sex, socioeconomic status, area, region and survey year. **Socioeconomic status (SES):**^a^low SES vs. medium and high SES, ^b^medium SES vs. high SES. **Region:**^a^North vs. Center and South, ^b^Center vs. Mexico City, ^c^Mexico City vs South, ^d^ Center vs South. **Survey year:**^**a**^2006 vs 2012, ^b^2012 vs 2016, ^c^2006 vs 2016

By SES, we found that those of lower levels obtained higher scores (*p* < 0.001) in all three surveys compared to those of medium and higher levels. By area of residence, we observed that urban areas scored significantly lower (*p* < 0.001) than rural areas in all 3 years of the survey.

By region, we found significantly lower scores in Northern Mexico and in Mexico City compared to the Center and South of the country (*p* < 0.05) in all survey years; the South scored substantially higher than other regions throughout the 3 years.

## Discussion

Based on the Healthy Eating Index (HEI-2015), our study identified the dietary patterns of the Mexican adults aged 20–59 years who participated in the 2006, 2012 and 2016 National Health and Nutrition Surveys (*ENSANUTs*).

Despite the higher scores observed in 2006 and 2016 compared to 2012, diet quality in both years was poor (equivalent to less than 50 points on a scale of 0 to 100). Since diet quality is related to non-communicable diseases such as obesity, diabetes mellitus II and cancer [12, 37], these scores were likely associated with the high prevalence of these diseases in Mexico.

Our analysis by sociodemographic characteristic indicated that diet differed according to age, sex and SES as well as to area and region of residence. The survey population between 40 and 59 years, women, those of lower SES and those living in rural areas and in Southern Mexico generally displayed a higher-quality diet, a trend which continued over time. National-level studies in other countries have documented that women and older adults have higher-quality diets than men [8, 38, 39] and younger adults [8, 39], respectively.

As regards the socioeconomic analysis, our results are the opposite of what has been described at the national level for high-income countries. According to previous studies, populations of higher SES have healthier diets than those of lower SES [14, 40]. We found low diet quality in all three levels of SES.

Our results brought to light several HEI-2015 components in which trends were the opposite of the general results observed by sociodemographic characteristic. For example, compared to women, men achieved higher scores for fatty acids and, in 2016, higher scores for saturated fats. These results are consistent with a previous study that evaluated the HEI-2015 based on a multiethnic cohort of adults, finding that men scored higher than women for this component [41]. In our study, this can be explained by the fact that, compared to women, men derived a lower percentage of their total energy intake from saturated fats although they consumed more saturated fats in grams. They also consumed high proportions of carbohydrates and proteins which contributed to their total energy intake (data not shown).

The urban population achieved higher scores for dairy products and proteins than the rural population, consistent with the results reported by other studies of Mexican adults. Those studies indicate that urban areas are associated with higher consumption of dairy products in addition to meat or animal products [12, 42]. This may be related to the greater availability of food in urban as compared to rural areas [43–45]. Studies of food consumption preferences in Mexico have documented that corn consumption decreases as diets become more varied [46]. We obtained similar results when comparing consumption in urban and rural areas.

With regard to sociodemographic characteristics, we observed that individuals of higher SES had higher scores for dairy products, proteins, refined grains and total fruits. Systematic reviews of dietary patterns among middle- and low-income countries as well as in European adults have documented that those of higher SES consume more fruits and refined grains [9, 40]. Studies in Mexico have shown that a higher SES is associated with increased consumption of dairy products and fruits along with vegetables and non-processed meats [14, 42, 47, 48]. These results are possibly linked to the costs of these foods, more affordable for those of higher SES (seen as a proxy for higher-income level) [44, 48]. The preceding results may be related to the fact that the population with higher SES also has lower scores for saturated fats, indicating that various dietary aspects should be monitored. Evidence indicates that a higher educational level in adults is associated with greater intake of vegetables and fruits and a lower intake of red meat [49]. Although our analysis did not include this variable, it is reasonable to expect that higher socioeconomic status (SES) reflects a higher educational level of the head of household [50].

By region, we observed higher scores in Southern Mexico for whole and refined grains. The North, meanwhile, had lower scores than other regions for total and whole fruits as well as whole grains, but higher scores for total proteins. These results are similar to those of studies that have compared dietary patterns among regions. The North has recorded lower levels of fruit and vegetable consumption but higher intake of meat and animal products [42, 47] as well as legumes [14] (which in this case form part of the total protein group).

In analyses carried out using the original HEI-2015 cutoff points for sodium, saturated fats and added sugar, we observed high scores, close to the maximum (Additional file 2: Fig. S1). These results are not consistent with those of other studies, as 64% of the adult Mexican population has been found to exceed the recommendation that < 10% of total energy should be derived from added sugars. Furthermore, 50% of this population exceeds the same recommendation with respect to saturated fats [51]. We assume that high scores were recorded for these three components because the scoring range for the original HEI-2015 used in our study was somewhat broad and permissive. This is reflected in the fact that for individuals to obtain a score of 0 on the Index, their intakes of added sugars and saturated fat need to exceed 26 and 16%, respectively, of their total energy consumption. These proportions are higher than those recommended by the WHO [31], which is the standard commonly used in Mexico.

The results of the analyses of these three components adhere more closely to those described in other studies. This is attributable to the fact that we changed the cutoff points for the minimum scores of zero (≥10% for added sugar, ≥10% for saturated fat and ≥ 2.0 g per day for sodium) and the cutoff point for the maximum score for sodium (≤1.1 g per day) (Additional file 1: Table S1).

Our study had several limitations. The first concerned the fact that the food frequency questionnaire used by the *ENSANUTs* did not fully capture the consumption of sodium. Accordingly, our analysis considered only sodium intrinsic to the foods studied and the average amount of salt added during the food preparation process included in the questionnaire, but omitted salt added to food already prepared and ready to eat.

Another limitation was related to the fact that the 2006 and 2012 *ENSANUTs* differed from the 2016 *ENSANUTMC* as to the time period during which the surveys were conducted; results may have been affected by the seasonal consumption of beverages and thus by the questionable comparability of survey data. Nonetheless, we feel that the scores for HEI-2015 components were unaffected by seasonality. Moreover, it was not our objective to analyze the contribution of specific micronutrients such as vitamins.

Salient among the strengths of this study were the size and type of our sample, being representative nationally as well as by areas and regions. An additional strength concerns the fact that our work constitutes one of the first assessments of diet quality in Mexico that describes the dietary patterns of Mexican adults using a methodology comparable over time. This study therefore provides evidence regarding trends in the diet quality of the population and contributes to the establishment of new study hypotheses as well as to the creation of targeted public policies for nutrition.

## Conclusions

In this study, we identified the trends in the consumption patterns of Mexican adults, obtaining useful insights into the diet quality of various population subgroups in the country. The resulting patterns show an overall decline in the diet quality of both age groups analyzed over the 3 years of our study. Most notably among the adults in the younger age group, the progressive penetration of harmful foods into the daily diet clearly shows the impact that socioeconomic factors have exerted on diet quality in Mexico over the past years.

In conclusion, the consumption patterns of the Mexican adult population have indicated poor-quality diets throughout the years. Furthermore, our analysis of adult consumption from 2006 to 2016 brings to light the persistence of notorious disparities among sociodemographic strata.

Our results can serve as a set of practical recommendations on specific aspects of diet quality for the adult population. In those cases where components achieved scores far from satisfactory, public-health messages could emphasize the importance of improving diet quality for the public. The results of our study can also inform the design of public policies oriented at reducing nutritional inequalities.

Diet quality, as related to indicators for chronic non-communicable diseases, has been suboptimal in recent years for the Mexican adult population. It is therefore important to contemplate undertaking further evaluations of diet quality over time to elucidate changes linked to disease and to monitor the adherence of the population to dietary guidelines and recommendations.

## Supplementary information

**Additional file 1: Table S1.** Components and scoring standards of the Healthy Eating Index-2015.

**Additional file 2: Figure S1.** Original HEI-2015 scores (medians) vs. maximum scores, by component and survey year.

## Data Availability

The datasets used and/or analyzed during the current study are available from the corresponding author on reasonable request.

## Abbreviations

HEI: Healthy Eating Index
SES: Socioeconomic Status
*ENSANUT*: National Health and Nutrition Survey
*ENSANUTMC*: Half-Way National Health and Nutrition Survey
SFFQ: Semi-quantitative food-frequency questionnaire
*INSP*: National Institute of Public Health
BMR: Basal metabolic rate
TEI: Total energy intake
WHO: World Health Organization
BMI: Body Mass Index
FPED: Food Patterns Equivalents Database
