# Supplementary material for: Trends in the dietary patterns of Mexican adults by sociodemographic characteristics
Source: Nutr J. 2020 May 27;19:51. doi: 10.1186/s12937-020-00568-2 (PMC7254758; doi:10.1186/s12937-020-00568-2)
Supplement: Supplementary file 1 — Additional file 1: Table S1. Components and scoring standards of the Healthy Eating Index-2015. [file 12937_2020_568_MOESM1_ESM.docx]

Supplementary table. Healthy Eating Index-2015 components and standards for scoring^1^

| Component | Maximum points | Standard for maximum score | Standard for minimum score of zero |
| --- | --- | --- | --- |
| Adequacy: |  |  |  |
| Total Fruits^2^  Whole Fruits^3^  Total Vegetables^4^  Greens and Beens^4^  Whole Grains  Dairy ^5^  Total Protein Foods^6^  Seafood and Plant Protein^6,7^  Fatty Acids^8^ | 5  5  5  5  10  10  5  5  10 | ≥0.8 cup equiv. per 1,000 kcal  ≥0.4 cup equiv. per 1,000 kcal  ≥1.1 cup equiv. per 1,000 kcal  ≥0.2 cup equiv. per 1,000 kcal  ≥1.5 oz equiv. per 1,000 kcal  ≥1.3 cup equiv. per 1,000 kcal  ≥2.5 oz equiv. per 1,000 kcal  ≥0.8 oz equiv. per 1,000 kcal  (PUFAs + MUFAs) / SFAs ≥2.5 | No Fruit  No Whole Fruit  No Vegetables  No Dark Green Vegetables or Legumes  No Whole Grains  No Dairy  No Protein Foods  No Seafood or Plant Proteins  (PUFAs + MUFAs) / SFAs ≤1.2 |
| Moderation: |  |  |  |
| Refined Grains  Sodium  Added Sugars  Saturated Fats | 10  10  10  10 | ≤1.8 oz equiv. per 1,000 kcal  ≤1.1 gram per day ^a^  ≤6.5% of energy  ≤8% of energy | ≥4.3 oz equiv. per 1,000 kcal  ≥2.0 gram per day ^a^  ≥10% of energy ^a^  ≥10% of energy ^a^ |

1: Intakes between the minimum and maximum standards are scored proportionately.

2: Includes 100% fruit juice.

3: Includes all forms except juice.

4: Includes legumes (beans and peas).

5: Includes all milk products, such as fluid milk, yogurt and cheese, and fortified soy beverages.

6: Includes legumes (beans and peas)

7: Includes seafood, nuts, seeds, soy products (other than beverages), and legumes (beans and peas).

8: Ratio of poly- and monosaturated fatty acids (PUFAs and MUFAs) to saturated fatty acids (SFAs)

^a^ Proposed modification to the cut-off points according to Mexican food guides
